# Supplementary material for: Assessment of public health impact of work-related asthma
Source: BMC Med Res Methodol. 2012 Mar 5;12:22. doi: 10.1186/1471-2288-12-22 (PMC3339512; doi:10.1186/1471-2288-12-22)
Supplement: Additional file 1 — WHO Subregional country grouping. [file 1471-2288-12-22-S1.DOC]

9 November 2011

**Assessment of public health impact of work-related asthma**

Maritta S. Jaakkola^1^, MD, PhD and Jouni J.K. Jaakkola^2^, MD, DSc,PhD

^1^Corresponding author, Respiratory Medicine Unit, Center for Environmental and Respiratory Health Research and Institute of Clinical Medicine, University of Oulu, P.O. Box 5000, 90014 Oulu, Finland, and Oulu University Hospital, Finland, e-mail: maritta.jaakkola@oulu.fi

^2^Center for Environmental and Respiratory Health Research and Institute of Health Sciences, University of Oulu, P.O. Box 5000, 90014 Oulu, Finland, e-mail: jouni.jaakkola@oulu.fi

**Abstract**

(350 words)

**Background:** Asthma is among the most common chronic diseases in working-aged populations and occupational exposures are important causal agents. Our aims were to evaluate the best methods to assess occurrence, public health impact, and burden to society related to occupational or work-related asthma and to achieve comparable estimates for different populations.

**Methods:** We addressed three central questions: **1: What is the best method to assess the occurrence of occupational asthma?** We evaluated: 1) assessment of the occurrence of occupational asthma *per se*, and 2) assessment of adult-onset asthma and the population attributable fractions due to specific occupational exposures. **2: What are the best methods to assess public health impact and burden to society related to occupational or work-related asthma?** We evaluated methods based on assessment of excess burden of disease due to specific occupational exposures. **3: How to achieve comparable estimates for different populations?** We evaluated comparability of estimates of occurrence and burden attributable to occupational asthma based on different methods.

**Results:** Assessment of the occurrence of occupational asthma *per se* can be used in countries with good coverage of the identification system for occupational asthma, i.e. countries with well-functioning occupational health services. Assessment based on adult-onset asthma and population attributable fractions due to specific occupational exposures is a good approach to estimate the occurrence of occupational asthma at the population level. For assessment of public health impact from work-related asthma we recommend assessing excess burden of disease due to specific occupational exposures, including excess incidence of asthma complemented by an assessment of disability from it. International comparability of estimates can be best achieved by methods based on population attributable fractions.

**Conclusions:** Public health impact assessment for occupational asthma is central in prevention and health policy planning and could be improved by purposeful development of methods for assessing health benefits from preventive actions. Registry-based methods are suitable for evaluating time-trends of occurrence at a given population but for international comparisons they face serious limitations. Assessment of excess burden of disease due to specific occupational exposure is a useful measure, when there is valid information on population exposure and attributable fractions.

**Background**

Asthma is among the most common chronic diseases in working-aged populations and in this age group, occupational exposures have been suggested to be important causal agents [1-4]. In developing countries the workforces probably have even more extensive occupational exposures than in high-income countries, but smaller figures of the occurrence of occupational asthma have been reported, suggesting that there is likely to be a problem of underdetection [5, 6] and that the methodology to assess the occurrence is not optimal. According to current understanding, occupational asthma may be healed if the person is removed from the specific causal exposure early enough [7], but unfortunately this rarely happens, as the detection and treatment of the disease are often delayed. Thus, occupational asthma is usually a chronic condition that is accompanied by disability, reduced workability and increased health care costs. It is practically always accompanied by economic losses to the individual worker as well as to the society [8].

Despite being the most common occupational lung disease worldwide, with pneumoconioses also common in the developing countries, there is little data on international comparisons of occurrence of occupational asthma or its public health impact. This is partly explained by difficulties in the methods used for assessing these. The methods that have been used are influenced by many country-specific factors, such as detection and diagnostic procedures, as well as workers compensation practices and coverage. There is a need to develop the methodology for assessing the impact of work-related asthma to provide a comprehensive picture for the purposes of planning preventive actions and health policy.

The purpose of this article is to provide a framework for methods to be used for assessing public health impact of work-related asthma. This includes presenting the methods (with focus on new methods), considering their strengths and limitations, and suggesting extension of applying these methods in future research of work-related asthma. We include evaluation of suitability of these methods for different study questions and purposes for doing the assessment.

The specific aims of this paper are to address the following questions:

1. What is the best method to assess occurrence of occupational or work-related asthma?
2. What are the best methods to assess public health impact and burden to society related to occupational or work-related asthma?
3. How to achieve comparable estimates for different populations including people in the developing countries?

**Methods**We addressed these three questions by evaluating suitability of the central epidemiologic measures of occurrence (prevalence, incidence rate), effect (risk ratio, incidence rate ratio), public health impact (attributable fraction, population attributable fraction) and burden of disease in the context of occupational exposures and asthma. We considered the questions and issues related to these methods from both theoretical and empirical perspectives, the latter by taking examples from the existing literature. Selection of these examples was based on using recent studies that provided data for international comparison and were useful in illustrating the strengths and limitations of the methods used.

For our analysis we identified three main types of relations between work and asthma: work and development of asthma, work and aggravation of symptoms and signs of asthma, and asthma and workability. The definitions of these may vary across countries, for example because of the legal issues related to asthma in the workplace, but we propose the following terms and definitions that are given in Table 1. Their interrelations are shown in Figure 1:

This paper will focus mainly on occupational asthma and to some extent on work-aggravated asthma, because the preventive questions related to public health impact of asthma in the workplace are mainly linked to these. Both of them may affect workability of the person, but asthma and workability is a larger question as it involves the whole working asthmatic population.

**Results and Discussion**

**Question 1: What is the best method to assess the occurrence of occupational asthma?**

Theoretically, two epidemiologic approaches can be used to assess the occurrence of occupational asthma in a given population:

1. Assessment of the occurrence of occupational asthma *per se*.
2. Assessment of adult-onset asthma and the attributable fractions and population attributable fractions due to specific occupational exposures.

The former approach involves identifying individuals with diagnosed occupational asthma in a specified population, whereas the latter gives an estimate of the occurrence of occupational asthma at the population level, but does not identify individuals with occupational asthma.

***Assessment of the occurrence of occupational asthma per se***

For assessing the occurrence of occupational asthma in a given population *per se* one needs an estimate of the numerator representing the cases of occupational asthma and the denominator representing the population at risk which produces the cases, expressed in person-time.

For this calculation the numerator should be formed of verified cases of occupational asthma. Whether these should be incident (new) cases of occupational asthma or whether prevalent cases could be used depends on the *purpose* of the estimation. Incident cases are more suitable than prevalent cases when assessing effects of occupational exposures on the etiology of asthma and for predicting future trends of public health burden from occupational asthma. Assessment of attributable fractions and burden of disease is usually based on incident data. However, prevalent cases of asthma may be relevant when assessing total burden to public health from current cases, i.e. when assessing burden from increased symptoms and health care utilization from asthma, because both prevalent and incident cases contribute to the burden due to illness, disability, health care costs and other consequences of asthma. However, it is not always easy to decide whether a case of occupational asthma is incident or prevalent. This difficulty is illustrated when considering a person who has had childhood asthma with a long intermittent period without asthma and recurrence of the disease in relation to a specific occupational exposure. In the opinion of the authors, this subject should be counted as having incident occupational asthma, if the disease would not have recurred in the absence of the specific workplace exposure. This view is based on definition of causality using counterfactual statements [9]. According to counterfactual reasoning, the statement that John's occupational exposure caused his asthma is equivalent to saying that had John not experienced the occupational exposure he would not have developed asthma. Accepting this we see that for causality in adulthood it is irrelevant whether or not John had asthma in childhood from which he had recovered. More relevant is that John is without asthma when encountering the exposure of interest. Similar reasoning can be applied to groups of individuals and probabilities of developing asthma among exposed and unexposed.

The choice of the right denominator is a difficult task and is also dependent on the purpose of the assessment or study question to be addressed. For assessing the incidence of occupational asthma, the denominator should be person-years at risk of getting occupational asthma in the population in which the occurrence is assessed. This is easily calculated if we have a specific study population followed for answering the question on occurrence of occupational asthma, but needs more consideration when using existing population registries. At the population level, the right population for assessing incidence of occupational asthma is adult population that has ever been at work, as occupational asthma may be detected even after the person has quit his/her job, although in such a case the relevant time-period at risk may be limited to a few years. Sometimes only those in certain ‘high-risk’ occupational groups are included as being at risk, but as new causes of occupational asthma are constantly identified even in workforces that have traditionally not been considered as risk-occupations, this approach may not be valid. When assessing occupational asthma incidence for certain occupational groups and in relation to specific exposures, people ever exposed to those specific exposures or working in those specific occupations is the relevant denominator by the same logic.

When assessing the prevalence of occupational asthma, the total adult population at certain point in time (for estimating prevalence) and certain time period (for estimating period prevalence) can be used as the denominator, as the purpose of such assessment is usually related to current burden to society from ill-health and heath care burden from all the existing cases.

The issues related to the accuracy and comparability of this type of assessment of occurrence of occupational asthma include questions on

- How to define occupational asthma?
- How should occupational asthma be verified?
- What is the coverage of the identification system for occupational asthma?
- What is the access to (occupational) health services?
- What are the workers’ compensation practices?
- How does the whole social security system influence all these?

The definitions of occupational asthma vary from country to country as do the identification and diagnostic procedures. Countries with well developed occupational health care systems tend to have a broader coverage and this is likely to lead to higher estimates of occurrence. Well-functioning workers’ compensation system may enhance detection of and reduce ill-health from occupational asthma, but on the other hand the compensation system may influence the diagnostic procedures and decisions so that cases with occupational asthma that would not be compensated might not be diagnosed at all. An example of this could be irritant-induced asthma for which the diagnostic procedures are less standardized than for hypersensitivity-type of occupational asthma and may go undetected if the compensation system requires very specific diagnostic tests, such as specific bronchial inhalation challenges [10]. On the other hand, if there are poor compensation and social security systems for those who develop occupational asthma, diseased workers may not seek medical help and continue working under exposure until the point where their asthma has become severe and causes severe disability.

To demonstrate some issues affecting the estimates based on this assessment approach, consider a comparison of registry data from two regions. The other data come from Finland, a high-income country with high quality and coverage of occupational health services and mandatory workers’ compensation for employees. The other data come from West Midlands in UK, also a high-income country but with larger socio-economic differences and a substantially smaller proportion of the workforce with appropriate occupational health services. The population size of these two areas is quite similar: 5 326 314 inhabitants in Finland in 2009 and 5 267 308 inhabitants in West Midlands in 2002 [11, 12]. The working-age population (defined as 20-64 years old adults for the purposes of these calculations) in West Midlands was slightly larger (3 061 210) than in Finland (2 839 686). The number of new cases of occupational asthma reported to the Finnish Register of Occupational Diseases between 1983 and 2002 [13] is presented in Figure 2 and that reported to the SHIELD Register in West Midlands during 1980 to 2002 [14, 15] is presented in Figure 3. The beginning of the follow-up period showed an increasing trend in both countries, but the numbers seemed to stabilize around 1989, perhaps due to more standardized diagnostic procedures and reporting practices. The number of new cases in Finland during this period was between 270 and 400 per year, and that in West Midlands 70-140 annually. Calculated based on the mean number of new cases per year during 1989-2002, the average incidence rate of occupational asthma was approximately 0.10 per 1000 person-years in Finland and 0.03 per 1000 person-years in West Midlands. For comparison, the incidence rate of adult-onset asthma in the working age population was in Finland in the late 1990s 0.9 per 1000 person-years estimated in a population-based incident case-control study [16]. The substantially larger incidence of occupational asthma in Finland is surprising in the light of a slightly smaller working-age population and West Midlands being an area with more traditional industry. The factors that may explain the observed difference are likely to be related to the occupational health services coverage and the fact that in Finland the reporting of new cases to the registry is mandatory, while in West-Midlands it is voluntary and based mainly on one occupational lung disease specialist centre. In Finland the diagnostic criteria were stricter requiring usually a specific inhalation challenge, while in West Midlands evidence from serial PEF measurements was accepted as a confirmatory test. This fact could be expected to decrease the rate of diagnosed cases in Finland compared to West Midlands, but as the results show an opposite trend, this further emphasizes the differences in coverage of health care services, compensation systems and reporting systems.

This example demonstrates that even if certain factors that influence the figures in registries could be better standardized by international consensus agreements, for example the definition of occupational asthma and the diagnostic criteria, many factors affecting the results are difficult to take into account, e.g. access to occupational health care and compensation practices. Thus, national or regional registries that receive their data from the routine health care practices provide useful information for assessing trends in occupational asthma over time at national or regional level, but they do not provide very useful data for international comparisons. The absolute values are highly influenced by the health care and social security systems, so these figures may not be of value for calculating public health burden from occupational asthma.

***Assessment based on the occurrence of adult-onset asthma and attributable fractions and population attributable fractions due to specific occupational exposures***

For assessing the occurrence of occupational asthma and its public health impact based on attributable fractions and population attributable fractions due to specific occupational exposures, the following estimates are needed:

1. An effect estimate for a specific or all occupational exposures, in the form of incidence rate ratio (IRR), and
2. An estimate of attributable fraction (AF) calculated based on this, and
3. An estimate of population attributable fraction (PAF) calculated based on AF and the prevalence of occupational exposure(s) of interest in the population for which the assessment is made (P_e_).

Incident rate ratio gives an estimate of the risk of developing asthma in relation to the exposure of interest and can be calculated according to the formula given in Table 2.

Odds ratio (OR) can be used to substitute IRR with the following considerations. ORs from incident case-control studies with density sampling of controls are unbiased estimates of IRR, whereas ORs from cohort studies and prevalent case-control studies are reasonable approximates for IRRs when the prevalence of adult asthma is <10%, which is true in most parts of the world.

Attributable fraction can be calculated by the formulas shown in Table 2. Attributable fraction assesses the impact of exposure by measuring its contribution to the total incidence under exposure [17], so it is used to estimate the proportion of exposed cases for whom the disease could be attributed to the exposure of interest. This fraction is sometimes interpreted as the probability that exposure caused the case and called the etiologic fraction. However, such an interpretation should be applied with caution, remembering that this approach applies to a population rather than to an individual case. When investigating a potential case of occupational asthma in a specialist clinical center, it should be remembered that the referral process usually works towards increasing the probability that exposure caused the case. For example, detecting work-related pattern of asthma symptoms increases the probability that occupational exposure caused the disease in this specific case and also increases the likelihood of referral to the specialist center for further investigations.

The methodological issues related to the accuracy and comparability of assessment based on attributable fraction include the following questions:

- Is the estimate of exposure used for assessing the effect valid?
- Is the effect estimate valid?

When assessing attributable fraction, the estimate of exposure should be accurate in terms of giving a valid effect estimate, but it does not need to be representative of the entire population. The latter is needed when assessing the population attributable fraction. However, the effect is likely to vary according to the quantity of exposure, so assessment of the exposure distribution is preferable over dichotomous exposure assessment (which just compares all exposed to the unexposed). Having valid quantitative exposure estimate would make it possible to estimate the risk function according to different exposure levels, giving a better understanding of the risk for different quantities of exposure. To ensure a valid effect estimate, this should be based on a high quality study or on meta-analysis if enough high quality data is available to carry out such analysis. High quality study should be based on incident cases and be free of any majors biases and confounding and it would be valuable if a quantitative assessment according to the exposure level had been conducted. Assessment of attributable fraction is comparable between populations that have similar exposure (both qualitatively and quantitatively), so this assessment is less dependent on country-specific health care and insurance systems.

Assessment of population attributable fraction (PAF) requires an estimate of the prevalence of the occupational exposure(s) of interest for the population of interest (P_e_) and an estimate of AF or IRR. The formulas for calculation of PAF are also given in Table 2. Population attributable fraction is defined as reduction in incidence that would be achieved if the population were entirely unexposed [17]. In public health it is interpreted as the proportion of cases in the population that could be prevented if exposure would be reduced to zero [18, 19].

The final step in assessing occurrence of occupational asthma based on estimating population attributable fraction due to specific occupational exposure(s) is calculating Excess burden of disease (EBD). Excess number of cases attributable to specific occupational exposures can be calculated by the formula shown in Table 2.

The issues related to the accuracy and comparability of the assessment of occurrence of occupational asthma based on population attributable fraction and excess burden of disease include the following questions:

- Is the estimate of exposure valid and representative of the population?
- Is the effect estimate valid?
- Is the estimate of incidence of asthma valid for the population of interest?

The estimate of exposure should be valid in relation to the effect estimate used and it should also be representative of the exposure, preferably exposure distribution, of the population for which the assessment is made. Again, the effect estimate should be from a high quality study or meta-analysis if there is enough high quality data available. And again, a risk function according to different exposure levels would be preferable over a risk ratio related to dichotomous exposure. The comparability of values given by this method of assessment for different populations depends on the similarity of exposures, both qualitatively and quantitatively. When assessing excess burden of disease another issue of importance is how to get a valid estimate of the incidence of asthma for the population(s) for which the assessment is made. This may be attained from a high-quality study or in some countries from existing registries. As an example of the latter, in Finland the National Institute of Social Security keeps a register on all cases of asthma who receive special reimbursement for asthma medications. Asthmatics usually receive this right and are registered about six months after their diagnosis is made, so the registry reflects rather well the incidence of asthma. However, it is not always possible to be sure that the case registered is new and that the case has asthma rather than severe COPD, so for a high quality study the medical records of the cases should be checked.

In summary, the method based on assessment of population attributable fraction and excess burden of disease provides good and comparable estimates of excess incidence (and public health impact) due to specific occupational exposures and specific occupational groups. This approach is not much affected by country-specific practices, such as occupational health access and workers compensation system, as is the assessment based on identifying cases diagnosed with occupational asthma *per se*.

However, the assessment of burden of disease from occupational exposures overall is accompanied by further methodological issues. The major one is the decision on which occupational groups or occupational exposures should be included in the exposed category and which occupations should form the unexposed reference category. Usually administrative employees are used as the reference group, as they do not have any heavy industrial-type of exposures, but more recent research has identified factors in office-type indoor spaces that seem to be determinants of adult-onset asthma [20-22], so this may not be an optimal reference group. Including all those not currently at work in the reference category may be especially problematic, as some people may be unemployed because of previous work-related symptoms. If all other occupations than administrative work is included in the ‘exposed’, this will include many jobs that do not have any exposures that would be expected to induce asthma. Thus, this would dilute the effects of exposures with clear asthmagenic effect. If the decision about including occupations in the exposed category is based *a priori* on studies reporting an IRR or OR >1, it should be remembered that the choice of the reference category influences the risk estimate for the specific occupational groups. If a more restrictive criteria would be applied to include only occupations for which the lower 95% confidence interval of IRR or OR is >1, one should remember that the sample size of the original study influences the width of the confidence interval, and thus the significance of the findings. In addition, it is difficult to foresee where new asthmagenic exposures turn up, as new chemicals are introduced into the working life all the time.

**Question 2. What are the best methods to assess public health impact and burden to society related to occupational or work-related asthma?**

The question on the best methods to assess the public health impact and burden to society related to occupational or work-related asthma cannot be answered by one simple reply. Different approaches complement each other. The authors recommend that such assessment should include an assessment of excess burden of disease due to specific occupations or occupational exposures as well as assessment of other aspects of burden to society.

Assessment of excess burden of disease should include an assessment of excess incidence of asthma due to occupational exposures, but this should be complemented by an assessment of disability related to occupational asthma. This could follow the approach used by WHO in environmental burden of disease assessments that include an estimation of excess disability adjusted life years (DALYs) [18, 23]. While mortality is a less useful measure of burden in the case of asthma than in the case of some other diseases, such as cancer, due to a less fatal natural history and rather good treatments currently available for asthma, DALYs are likely to be useful measures for occupational asthma. DALYs are the sum of the years of life lost due to premature mortality (YLL) and the years of healthy life lost due to disability (YLD) in incident cases of the health condition [24], calculated by the formula given in Table 2. DALYs are useful for estimating potential benefits that could be achieved in the population from preventive actions. The comparability of this measure between different populations is reasonably good, if it can be assumed that the severity of occupational asthma is similar in different populations.

To get a full picture of the public health impact and burden to society it is important to complement the PAF-based assessment of excess cases and DALYs with assessment of other consequences of occupational asthma, including health care use, sickness absenteeism, unemployment and economic costs [25]. The PAF-based assessment approach (utilizing prevalent cases) could be applied to health care use and sickness absenteeism, when the total burden from asthma is known. This would give, for example, the excess use of health care for asthma attributable to occupational exposures However, it should be remembered that these as well as economic costs are influenced by country-specific factors, i.e. they depend on the health care and economic systems. Because of this also other methods should be applied to get a complete picture.

Assessment of economic costs is an important exercise to see how much occupational asthma, which is a disease condition that could be prevented or at least minimized with good control of harmful occupational exposures in the workplaces, costs to the diseased individuals, their employers and the whole society. The authors recommend that an assessment of costs should include at least the following elements:

- costs from diagnostic tests
- cost from treatments
- costs from sickness leaves
- costs from re-education
- costs from unemployment
- costs to the employer from recruitment and training of new workers
- costs from loss of productivity and innovation

A recent example of calculating the costs from health care, sickness absences and compensation due to occupational asthma in UK was published in 2011 [26]. The costs from unemployment, recruitment and training of new workers and loss of productivity form large sums of money that are often not included in the health economic calculations and should get more attention in the future.

**Question 3: How to achieve comparable estimates for different populations including people in the developing countries?**

Comparability of the estimates of occurrence of and burden attributable to occupational asthma has been discussed already in the context of questions 1 and 2. Here we present two examples of applying population attributable fractions to assess burden of disease from workplace exposures with international comparisons to demonstrate the benefits as well as limitations of such approach.

The first study focused on assessing the public health impact of secondhand smoke (SHS) in the workplace with the aim to estimate how much benefit could be achieved by introducing smoke-free workplace legislation throughout Europe [27]. Adult-onset asthma was one of the health outcomes included in the assessment and the effect estimate was obtained from a large population-based incident case control study from Finland [28]. The OR of adult-onset asthma related to workplace SHS exposure during the last 12 months was 2.16 (95% CI 1.26-3.72) and the study suggested that SHS can induce asthma through low-dose long-term irritant mechanism. Exposure data in the early 2000s for 14 European countries and USA were obtained from the European Community Respiratory Health Survey [29] and from a population-based follow-up study from Finland [30]. The results on PAFs of asthma from workplace SHS are presented in Table 3. They show that in countries with high exposure prevalence at the time, including Spain, Italy, the Netherlands, Belgium, Germany, and Ireland, as much as 15%-29% of adult-onset asthma could be prevented by reducing workplace SHS exposure to zero. In low exposure countries, such as Sweden and Finland, the corresponding PAFs were 1-6%. After the exposure data used in this analysis was collected, Ireland, Italy and UK have introduced smoke-free workplace legislations. This study demonstrates that the PAF-based assessment is useful for international comparisons. In the case of assessing burden related to a specific workplace exposure, the choise of the reference category is usually not problematic (here no SHS exposure at work nor at home during the past 12 months) as long as potential exposure at home is also taken into account.

The other example was an attempt by WHO to assess the global burden of non-malignant respiratory disease due to occupational airborne exposures [31]. One of the diseases included in this assessment was asthma. Estimates of relative risk were obtained from two studies looking at the risk of asthma in relation to a variety of occupations [1, 32] and the decision on which occupations were classified as at risk were based on these same two studies. It was assumed that persons currently employed in occupations with potential exposure to asthmagens estimates well persons ever exposed to potential asthmagens at work. Estimates of exposures were obtained based on the workforce data from the International Labour Organisation for 2002 [33]. The results on PAFs used for calculating excess mortality are presented in Table 4. The largest PAFs were observed in Africa (18-20%), Europe (apart from west) (18%), South-East Asia (18%) and Western Pacific (19%), while the lowest PAFs were observed in North America (11%) and Western Europe (11%). This study estimated that in 2000 a total of 38 200 excess deaths occurred in the world due to occupational asthma, and it contributed to a vast amount of DALYs, altogether 1,6 million (Table 2). The region with the largest DALYs lost due to occupational asthma were Southeast Asia WHO region D, including countries such as India and Bangladesh, with 356 000 DALYs, and Western Pacific region B, including countries such as China, Vietnam and Philippines, with 356 000 DALYs. Other areas with large burden included African countries, South American countries and Eastern Mediterranean region D. This article also demonstrates the usefulness of applying PAF-based assessment for international comparisons. However, when interpreting the results, it is important to be aware of the assumptions used in the models. For example in this study, the choise of the reference occupations was based on studies conducted in Western Europe, which may not be optimal for the other parts of the world.

**Conclusions**

Estimation of the occurrence of occupational asthma can be based on the assessment of occurrence of occupational asthma *per se* when the denominator is known, or based on the assessment of occurrence of adult-onset asthma combined with the assessment of population attributable fraction from specific occupational exposures. The former approach can be used in countries with good coverage of the identification system for occupational asthma, i.e. countries with well-functioning occupational health services. However, this approach is strongly influenced by country-specific differences in diagnostic practices, the health care system and the workers’ compensation system.

Population attributable fraction based approach gives good comparability between countries, if the estimates of exposure, effect and incidence of adult-onset asthma are of high quality. Under those conditions this approach is superior to the other approach. This method works well at population level and is useful for the purposes of assessing public health impact of occupational asthma and for planning health care and health policies.

Assessment of burden to society should include assessment of excess cases of occupational asthma based on population attributable fractions, complemented by an assessment of disability that develops as a consequence of occupational asthma, for example disability adjusted life years. This method provides comparability of the assessed burden for different populations, if the severity of occupational asthma is similar in them. However, country-dependent factors such as a possibility to stop working in the exposure conditions affect the severity of occupational asthma. Other consequences of occupational asthma that should be assessed include health care use, sickness absenteeism, unemployment and economic costs from recruiting and training new workers and loss of productivity and innovation.

Public health impact assessment is central in prevention and health policy planning and should be improved by purposeful development of the methods. Registry-based methods are suitable for evaluating time-trends of occurrence at a given population but for international comparisons they face serious limitations. Assessment of excess burden of disease due to specific occupational exposure is a useful measure, when there is valid information on population exposure and attributable fractions.

**References**

1. Karjalainen A, Kurppa K, Martikainen R, Kaulkka T, Karjalainen J: **Work is related to a substantial portion of adult-onset asthma indicence in the Finnish population.** *Am J Respir Crit Care Med* 2001; **164**: 565-568.

2. Jaakkola JJK, Piipari R, Jaakkola MS: **Occupation and asthma: A population-based incident case-control study.** *Am J Epidemiol* 2003; **158**: 981-987.

3. Kogevinas M, Zock JP, Jarvis D, et al: **Exposure to substances in the workplace and new-onset asthma: an international prospective population-based study (ECRHS-II).** *Lancet* 2007; **370**: 336-341.

4. Torén K, Blanc PD. **Asthma caused by occupational exposures is common – A systematic analysis of estimates of the population-attributable fraction**. *BMC Pulmonary Medicine* 2009; **9**: 7.

5. Jeebhay MF, Quirce S: **Occupational asthma in the developing and industrialized world: a review.** *Int J Tuberc Lung Dis* 2007; **11**: 122-133.

6. Chan-Yeung M, Becklake M: **Occupational lung disease: under-recognised, underestimated and poorly managed, even today.** *Int J Tuberc Lung Dis* 2007; **11**: 119.

7. Hendrick DJ, Burge PS. **Asthma**. In *Occupational Disorders of the Lung*. Edited by Hendrick D, Beckett WS, Burge PS, Churg A. London: Saunders/ Harcourt Publishers Limited; 2002: 33-76.

8. Nicholson PJ, Cullinan P, Burge PS, Boyle C: **Occupational asthma: Prevention, identification & management: Systematic review & recommendations**. London: British Occupational Health Research Foundation, 2010.

9. Lewis D: **Causation.** *The Journal of Philosophy* 1973, **70**:556-567 10. Cartier A, Bernstein IL, Burge PS et al.: [**Guidelines for bronchoprovocation on the investigation of occupational asthma. Report of the Subcommittee on Bronchoprovocation for Occupational Asthma.**](http://www.ncbi.nlm.nih.gov/pubmed/2809031) *J Allergy Clin Immunol* 1989; **84**: 823-829.

11. **Statistics Finland**. [http://www.stat.fi/tup/suoluk/suoluk_vaesto_en.html]

12. **Office for national statistics**. United Kingdom. [http://www.statistics.gov.uk/census2001/pyramids/pages/f.asp]

13. Piipari R, Keskinen H: **Agents causing occupational asthma in Finland in 1986-2002: cow epithelium bypassed by moulds from moistute-damaged buildings**. *Clin Exp Allergy* 2005; **35:** 1632-1637.

14. **Shield surveillance scheme for occupational asthma**. [<http://www.occupationalasthma.com/shield.aspx>]

15. Bakerly ND, Moore V, Vellore A, Jaakkola MS, Robertson AS, Burge PS: **Fifteen-year trends in occupational asthma: data from the Shield surveillance scheme.** *Occupational Medicine* 2008; **58**: 169-174.

16. Jaakkola MS, Ieromnimon A, Jaakkola JJK: **Are atopy and specific IgE to mites and molds important for adult asthma?** *J Allergy Clin Immunol* 2006; **117**: 642-648.

17. Rothman KJ, Greenland S, Lash TL: *Modern Epidemiology*, 3^rd^ edition. Philadelphia: Lippincott Williams & Wilkins; 2008.

18. Ezzati M, Lopez AD, Rodgers A, Murray CJL, eds. WHO | **Comparative Quantification of Health Risks: Global and regional burden of disease attributable to selected major risk factors**. Geneva: World Health Organization; 2004. [http://www.who.int/publications/cra/en/]

19. Miettinen OS: **Proportion of disease caused or prevented by a given exposure, trait or intervention*.*** *Am J Epidemiol* 1974;**99**:325-332.

20. Jaakkola MS, Nordman H, Piipari R, Uitti J, Laitinen J, Karjalainen A, Hahtola P, Jaakkola JJK: **Indoor dampness and molds and development of adult-onset asthma: A population-based incident case-control study.** *Environ Health Perspect* 2002; **110**: 543-547.

21. Jaakkola JJK, Ieromnimon A, Jaakkola MS: **Interior surface materials and asthma in adults: A population-based incident case-control study.** *Am J Epidemiol* 2006; **164**: 742-749.

22. Jaakkola MS, Jaakkola JJK: **Office work exposures and adult-onset asthma**. *Environ Health Perspect* 2007; **115**: 1007-1011.

23. Öberg M, Jaakkola MS**,** Woodward A, Peruga A, Prűss-Ustűn A: **Worldwide burden of disease from exposure to second-hand smoke: a retrospective analysis of data from 192 countries**. *Lancet* 2011; **377:**139-146.

24. Murray CJL, Salomon JA, Mathers CD, Lopez AD (eds.). *Summary measures of population health: concepts, ethics, measurement and applications.* Geneva: World Health Organization; 2002.

25. Blanc PD, Burney P, Janson C, Torén K: **The prevalence and predictors of respiratory-related work limitation and occupational disability in an international study**. *Chest* 2003; **124**: 1153-1159.

26. Ayres JG, Boyd R, Cowie H, Hurley JF. **Costs of occupational asthma in the UK**. *Thorax* 2011; **66**: 128-133.

27. Jaakkola MS, Jaakkola JJK: **Impact of smoke-free workplace legislation on exposures and health: Possibilities for prevention**. *Eur Respir J* 2006; **28**: 397-408.

28. Jaakkola MS, Piipari R, Jaakkola N, Jaakkola JJK: **Environmental tobacco smoke and adult-onset asthma: A population-based incident case-control study**. *Am J Public Health* 2003; **93**: 2055-2060.

29. Janson C, Chinn S, Jarvis D, Zock J-P, Torén K, Burney P: **Effect of passive smoking on respiratory symptoms, bronchial hyperresponsiveness, lung function, and total serum IgE in the European Community Respiratory Health Survey: a cross-sectional study***. Lancet* 2001; **358**:2103-2109.

30. Jousilahti P, Helakorpi S: **Prevalence of exposure to environmental tobacco smoke at work and at home: 15-year trends in Finland.** *Scand J Work Environ health* 2002; **28** (Suppl. 2): 16-20.

31. Driscoll T, Nelson DI, Steenland K, Leigh J, Concha-Barrientos M, Fingerhut M, Prűss-Ustűn A. **The global burden of non-malignant respiratory disease due to occupational airborne exposures.** *Am J Industrial Med* 2005; **48**: 432-445.

32. Kogevinas M, Anto J, Sunyer J, Tobias A, Kromhout H, Burney P: **Occupational asthma in Europe.** *Lancet* 1999; **353:** 1750-1754.

33. ILO. *Economically active population 1950-2010*, *4^th^ edition, rev. 2.* Geneva: International Labour Organization; 2002.

**Competing interests**

The authors declare no competing interests.

**Authors’ contributions**

Maritta S. Jaakkola has made substantial contributions to conception and design and analysis of data, she has drafted the manuscript and given approval for the final version to be published.

Jouni J.K. Jaakkola has made substantial contributions to conception and design, he has critically revised the manuscript and given approval for the final version to be published.

**Funding**

Academy of Finland, grants no. 129419 and 138691.

Table 1. Definitions of occupational asthma, work aggravated asthma, and asthma affecting workability, as used in this article

| **Occupational asthma** Adult-onset asthma for which a specific exposure or a combination of specific exposures in the workplace is the main cause |
| --- |
| **Work aggravated asthma**  Pre-existing asthma whose symptoms and manifestations are made worse by exposures in the workplace  This term has also been used for development of asthma for which occupational exposure /occupational exposures may be contributing factors, but not the main cause |
| **Asthma affecting workability**  Because of the asthma condition the person’s ability to perform his/her work tasks is reduced  In this case, the development of asthma may or may not be related to workplace exposures |

Table 2. Measures of effect and burden of disease

| **Measure and definition** | **Formula** |
| --- | --- |
| **Incidense rate ratio = IRR**  gives an estimate of the risk of developing asthma related to the exposure of interest in comparison to no exposure | IRR = IR_e_ / IR_0_  where IR_e_ is the incidence rate of asthma among the exposed and IR_0_ is the incidence rate of asthma among the unexposed |
| **Attributable fraction = AF**  estimates the proportion of exposed cases for whom the disease can be attributed to the exposure of interest | AF= (IRR-1) / IRR  or  AF= (IR_e_ –IR_0_) / IR_e_  where IR_e_ is the incidence rate of asthma among the exposed and IR_0_ is the incidence rate of asthma among the unexposed |
| **Population attributable fraction = PAF**  gives the reduction in incidence of disease that would be achieved if the population were entirely unexposed | PAF= P_e_ x AF  or  PAF= P_e_ x (IRR - 1)/ [P_e_ x (IRR - 1) + 1]  where P_e_ is an estimate of the prevalence of the occupational exposure(s) of interest for the population of interest |
| **Excess burden of disease = EBD**  gives the excess number of incident cases that can be attributed to the exposure of interest | EBD= PAF x totN_i_,  where PAF is the population attributable fraction due to the occupational exposure(s) of interest and totN_i_ is the total number of incident cases of adult-onset asthma |
| **Disability adjusted life years = DALY**  gives the sum of the number of years of life lost due to premature mortality and the number of years of healthy life lost due to disability in incident cases of the health condition | DALY = YLL + YLD  where YLL is the years of life lost due to premature mortality and YLD is the years of healthy life lost due to disability |

**Table 3. Population attributable fraction (PAF) for asthma attributable to workplace secondhand smoke (SHS) exposure for 14 European countries and USA.**

| **Country** | **PAF for asthma attributable to workplace SHS** |
| --- | --- |
| Spain | 17-29 |
| Italy | 16-22 |
| The Netherlands | 16-20 |
| Belgium | 15-16 |
| Germany | 13-16 |
| Ireland | 16 |
| France | 10-15 |
| UK | 6-13 |
| Switzerland | 11 |
| Norway | 10 |
| Iceland | 10 |
| Estonia | 7 |
| Sweden | 1-6 |
| Finland | 2 |
| USA | 17-28 |

Adapted from Jaakkola and Jaakkola 2006 [27]

**Table 4. Population attributable fractions (PAF), number of deaths and disability adjusted life years (DALYs) from asthma in 2000 attributable to workplace exposures by WHO region (see Appendix 1 online).**

| **Region** | **PAF** | **Number of deaths**  **(000s)** | **DALYs (000s)** |
| --- | --- | --- | --- |
| Afr-D | 18 | 1.6 | 90 |
| Afr-E | 20 | 3.1 | 141 |
| Amr-A | 11 | 0.6 | 51 |
| Amr-B | 13 | 1.3 | 125 |
| Amr-D | 13 | 0.2 | 19 |
| Emr-B | 12 | 0.4 | 21 |
| Emr-D | 16 | 2.2 | 100 |
| Eur-A | 11 | 1.4 | 55 |
| Eur-B | 18 | 2.0 | 43 |
| Eur-C | 18 | 2.8 | 41 |
| Sear-B | 18 | 3.8 | 70 |
| Sear-D | 18 | 11.8 | 476 |
| Wpr-A | 13 | 0.8 | 33 |
| Wpr-B | 19 | 6.2 | 356 |
| World | 17 | 38.2 | 1621 |

Adapted from Driscoll et al. 2005 [31].

**Table 5.** Summary of the methods to assess occurrence of occupational asthma and excess public health burden from occupational asthma: the method, its advantages and limitations and recommended applications

| **Method** | **Advantages** | **Limitations** | **Recommended applications** |
| --- | --- | --- | --- |
| Assessment of occurrence of diagnosed occupational asthma *per se*,  e.g. registries | Data collection takes place as part of the routine health care practices | Influenced heavily by country-specific differences in diagnostic practices, health care system, workers’ compensation system, reporting system | Can be used in countries with well-functioning occupational health services  Suitable for assessing national or regional trends over time  Not very useful for international comparisons |
| Assessment of occurrence (i.e. excess cases) based on population attributable fraction due to occupational exposures | Gives good and internationally comparable estimates of excess incidence attributable to occupational exposures  Not much affected by country-specific practices or health care or compensation systems | Needs valid, high quality estimates of exposure prevalence, health effect of exposure, and incidence of adult-onset asthma | Works well at population level  Suitable for assessing occurrence of occupational asthma for planning health care and health policies  Suitable for international comparisons |
| Assessment of public health burden measured as excess DALYs and other disability indicators based on population attributable fraction due to occupational exposures | Gives good and internationally comparable estimates of burden attributable to occupational exposures  Not much affected by country-specific practices or health care systems | Needs valid, high quality estimates of exposure prevalence, health effect of exposure, and total burden measured as DALYs and other disability indicators from asthma  Assumes that severity related to occupational asthma is similar to severity related to adult-onset asthma in general, and that the severity of occupational asthma is comparable between countries | Works well at population level  Suitable for assessing public health impact of occupational asthma for planning health care and health policies  Suitable for international comparisons |
| Other methods to assess health care use, sickness absenteeism, unemployment and economic costs due to occupational exposures | Complement the assessment of burden to society from work-related asthma | Influenced heavily by country-specific differences in health care system, workers’ compensation system, and social security system | Suitable for assessing national or regional trends over time  Gives complementary information on burden to society for planning health care and health policies  Not very useful for international comparisons |

**Figure 1.** illustrates the relation between asthma and work.

Work-related asthma includes occupational asthma and work-aggravated asthma. These may affect workability, but any asthma even if not related to work may influence the person’s workability.

**Figure 2.** New cases of occupational asthma in Finland between 1983-2002 reported to the Finnish Register of Occupational Diseases [13].

The colour indicates the causal agent. The population of Finland in 2002 was 5 326 314, 20-64 yr population 2 839 686, employed population 2 380 863.

**Figure 3.** New cases of occupational asthma in West-Midlands, UK, between 1980-2002 reported to the SHIELD register [14, 15].

The population of West-Midlands in 2001 was 5 267 308, 20-64 yr population 3 061 210, employed population 2 511 000.

**Appendix 1.** online

WHO Subregional country grouping

| **Region/ mortality stratum** | | **Countries** |
| --- | --- | --- |
| Afr | D | Algeria, Angola, Benin, Burkina Faso, Cameroon, Cape Verde, Chad, Comoros, Equatorial Guinea, Gabon, Gambia, Ghana, Guinea, Guinea-Bissau, Liberia, Madagascar, Mali, Mauritania, Mauritius, Niger, Nigeria, Sao Tome and Principe, Senegal, Seychelles, Sierra Leone, Togo. |
|  | E | Botswana, Burundi, Central African Republic, Congo, Côte d'Ivoire, Democratic Republic of the Congo, Eritrea, Ethiopia, Kenya, Lesotho, Malawi, Mozambique, Namibia, Rwanda, South Africa, Swaziland, Uganda, United Republic of Tanzania, Zambia, Zimbabwe. |
| Amr | A | Canada, Cuba, United States of America. |
|  | B | Antigua and Barbuda, Argentina, Bahamas, Barbados, Belize, Brazil, Chile, Colombia, Costa Rica, Dominica, Dominican Republic, El Salvador, Grenada, Guyana, Honduras, Jamaica, Mexico, Panama, Paraguay, Saint Kitts and Nevis, Saint Lucia, Saint Vincent and the Grenadines, Suriname, Trinidad and Tobago, Uruguay, Venezuela. |
|  | D | Bolivia, Ecuador, Guatemala, Haiti, Nicaragua, Peru. |
| Emr | B | Bahrain, Iran (Islamic Republic of), Jordan, Kuwait, Lebanon, Libyan Arab Jamahiriya, Oman, Qatar, Saudi Arabia, Syrian Arab Republic, Tunisia, United Arab Emirates. |
|  | D | Afghanistan, Djibouti, Egypt, Iraq, Morocco, Pakistan, Somalia, Sudan, Yemen. |
| Eur | A | Andorra, Austria, Belgium, Croatia, Cyprus, Czech Republic, Denmark, Finland, France, Germany, Greece, Iceland, Ireland, Israel, Italy, Luxembourg, Malta, Monaco, Netherlands, Norway, Portugal, San Marino, Slovenia, Spain, Sweden, Switzerland, United Kingdom. |
|  | B | Albania, Armenia, Azerbaijan, Bosnia and Herzegovina, Bulgaria, Georgia, Kyrgyzstan, Poland, Romania, Serbia and Montenegro, Slovakia, Tajikistan, The Former Yugoslav Republic of Macedonia, Turkey, Turkmenistan, Uzbekistan. |
|  | C | Belarus, Estonia, Hungary, Kazakhstan, Latvia, Lithuania, Republic of Moldova, Russian Federation, Ukraine. |
| Sear | B | Indonesia, Sri Lanka, Thailand. |
|  | D | Bangladesh, Bhutan, Democratic People's Republic of Korea, India, Maldives, Myanmar, Nepal, Timor Leste. |
| Wpr | A | Australia, Brunei Darussalam, Japan, New Zealand, Singapore. |
|  | B | Cambodia, China, Cook Islands, Fiji, Kiribati, Lao People's Democratic Republic, Malaysia, Marshall Islands, Micronesia (Federated States of), Mongolia, Nauru, Niue, Palau, Papua New Guinea, Philippines, Republic of Korea, Samoa, Solomon Islands, Tonga, Tuvalu, Vanuatu, Viet Nam |

Status 2004; WHO: World Health Organization; A=very low child mortality and very low adult mortality; B=low child mortality and low adult mortality; C=low child mortality and high adult mortality; D=high child mortality and high adult mortality; E=high child mortality and very high adult mortality. Afr=Africa. Amr=The Americas. Emr=Eastern Mediterranean. Eur=Europe. Sear=Southeast Asia. Wpr=Western Pacific Region. Adapted from World Health Organization. *The World Health Report 2002 - Reducing risks, promoting healthy life*. Geneva: World Health Organization, 2002. <http://www.who.int/whr/2002/en/index.html> (accessed November 8, 2011).
